# Assessment of burnout in medical students using the Maslach Burnout Inventory-Student Survey: a cross-sectional data analysis

**DOI:** 10.1186/s12909-020-02274-3

**Published:** 2020-10-21

**Authors:** Michael Obregon, Jessica Luo, Jarod Shelton, Terri Blevins, Martin MacDowell

**Affiliations:** 1 University of Illinois College of Medicine at Rockford, 1601 Parkview Ave, Rockford, IL 61107 USA; 2 Research Assistant Professor and Assistant Dean for Student Affairs and Academic Progress, University of Illinois College of Medicine at Rockford, Rockford, IL USA; 3 Research Professor and Associate Director Health Professions Education, Department of Family Medicine, National Center for Rural Health Professions, University of Illinois College of Medicine at Rockford, Rockford, IL USA

**Keywords:** Medicine, Students, Burnout, Maslach, MBI-SS, Motivation, Wellness

## Abstract

**Background:**

Medical student burnout can cause emotional and physical exhaustion and detachment. The objectives of this study were to evaluate burnout using the Maslach Burnout Inventory-Student Survey (MBI-SS), identify factors that may predict burnout, and assess wellness initiatives effectiveness at reducing burnout.

**Methods:**

The MBI-SS was administered to all medical students (Classes 2019 to 2022) at the University of Illinois College of Medicine (UICOM) from February to May 2019. Factor analysis and internal consistency of the MBI-SS were assessed. Mean MSBI-SS subscale scores for burnout were calculated for cynicism (CY), emotional exhaustion (EE), and academic efficacy (AE). Multiple regression analysis was used to identify student factors that may predict burnout.

**Results:**

A total of 273 (21.6%) UICOM students completed the survey and 110 (40.3%) respondents reported self-perceived burnout. MBI-SS subscale scores were significantly higher for CY and EE, and significantly lower for AE in students who reported suffering from burnout versus students who did not report burnout. Mean ± SD subscale scores for CY, EE, and AE in burnout students were 14.44 ± 5.59, 23.23 ± 4.74, and 24.81 ± 5.35, respectively. In comparison, mean ± SD subscale scores for CY, EE, and AE in non-burnout students were 7.59 ± 5.16, 14.96 ± 5.71, and 28.74 ± 3.21, respectively. Regression analysis denoted significant associations between burnout and being out-of-phase in the curriculum, the effectiveness of wellness initiatives, and strength of motivation for medical school (SMMS) in both the two- and three-dimensional MBI-SS models. Gender was significantly associated with burnout in only the two-dimensional model.

**Conclusions:**

Self-reported burnout in medical students at UICOM was validated using the MBI-SS. Being out-of-phase in the curriculum, being female, rating wellness initiatives as less effective, and demonstrating lower motivation for continued medical school education may be used as predictors of medical student burnout. This investigation may act as a guide for measuring burnout in medical student populations and how the implementation of wellness initiatives may ameliorate burnout.

## Background

Herbert Freudenberger first described burnout in 1974 as a ‘state of mental and physical exhaustion caused by one’s professional life [1]. The definition of burnout changes slightly depending on the population under investigation, yet, the underlying description explained by Freudenberger remains consistent, while its impact upon the health care system is ever evolving. The rate of self-reported burnout among physicians in the United States increased from 40 to 51% from 2013 to 2017, respectively [2]. Healthcare practitioner burnout may be characterized as ‘various degrees of emotional exhaustion, depersonalization and a low sense of personal accomplishment’ [3]. Physician burnout can decrease the accuracy of medical diagnoses, impede effective patient-physician communication, and increase the number of unnecessary medical procedures [4]. The annual economic impact of physician burnout has been estimated at $3.4 billion dollars with this loss expected to increase alongside worsening rates of burnout [5].

Numerous investigations have studied the origins of physician burnout and it has been reported that the phenomenon may begin manifesting as early as medical school [6, 7]. In fact, some studies suggest that approximately half of all medical students may be suffering from burnout before residency [8, 9]. The responsibilities of a medical student, in comparison to a resident or attending physician, are greatly limited due to their evolving knowledge base and clinical experience, nevertheless, medical students are expected to perform at a level that may seem unachievable. Corish estimated that the volume of medical knowledge has doubled every 73 days since 2010 [10]. Gradually, this new medical knowledge is integrated within the curriculum and students are expected to master the information for their United States Medical Licensing Examination (USMLE). The score a student receives on the USMLE, especially STEP 1, is directly correlated with their ability to matriculate in their desired specialty, and the stress associated with the examination has been shown to negatively impact a student’s wellbeing [11].

Aside from the stressors of classroom and the board exams, burnout has been linked with loneliness, depression, isolation, an inability to relate to one’s peers, and feelings of apathy and depersonalization [12]. Additionally, increased alcohol use/abuse, illicit drug use, and suicidal ideations have been associated with burnout in medical students [13–15]. These attributes have been shown to decrease the quality of care delivered to a patient and therefore, require immediate correction before a medical student is granted increased autonomy during residency.

Given the serious implications of medical student burnout, it is necessary to identify predictors that may influence the phenomenon. Acquiring a better understanding of the predictors of burnout may allow for resolutions to be developed that target the most severe causes. If the most significant causes are addressed, it may be possible to reduce the rate of burnout and improve the well-being of future physicians throughout their undergraduate and graduate medical education.

### Current study

The purpose of this investigation was to evaluate medical student burnout using the Maslach Burnout Inventory-Student Survey (MBI-SS) within a single medical school system in the United States. The research questions this paper sought to address are as follows: (RQ1) Can the MBI-SS be used to predict burnout in medical school students; (RQ2) Are there any associations between burnout and demographic factors that can be used to identify students at risk for developing burnout; and (RQ3) Are the use of wellness initiatives sufficient for reducing burnout among medical students?

## Methods

### Study design and setting

The study was approved by the Internal Review Board (IRB) at the University of Illinois at Rockford (UI-Rockford). Medical students from each class (M1-M4 and MD/PhD), totaling 1359 students, at the University of Illinois College of Medicine (UICOM) were asked to complete the cross-sectional survey. UICOM has four distinct campuses: Chicago, Peoria, Rockford, and Urbana-Champaign. MD/PhD students and all students at Urbana-Champaign were excluded (95 students) from the final analysis due to low response rates of < 6% and < 3%, respectively. The study was conducted from February 2019 to May 2019. The survey was hosted on the web-based application, Google Forms. A non-probability sampling method was used by sending several campus-wide emails to students requesting their voluntary participation. A non-probability sampling method was used to decrease the likelihood of selection bias for students who are less responsive to a single email. Four, $25 apparel items were offered as an incentive to complete the survey. Students were randomly selected for the incentive. Strengthening the reporting of observational (STROBE) guidelines for observational studies were followed throughout. The first page of the survey contained a statement of consent, agreeing to be included in the study. All 273 respondents who started the survey consented and completed the survey. No students refused consent.

### Measures

The survey (see Additional file 2) was a mix of demographic, career-oriented, medical specialty, MBI-SS, and SMMS questions. The 16-item MBI-SS is a validated tool used to measure burnout in student populations [16]. The MBI-SS (Mind Garden Inc., Menlo Park, CA) consists of three subscales to evaluate the different domains of burnout: emotional exhaustion (i.e. the draining of emotional resources because of demanding interpersonal contacts with others), cynicism (i.e. negative, callous, and cynical attitude towards the recipients of one’s care or services), and academic efficacy (i.e. tendency to evaluate one’s accomplishments with recipients negatively) [17]. Responses are provided on a 7-point Likert scale with higher values referencing more frequent occurrences. Higher scores for CY and EE while lower scores for AE suggest burnout. Item 13 was deleted from the MBI-SS based on prior investigations finding it to be ambivalent and unsound [18–20].

The 15-item Strength of Motivation for Medical School questionnaire (SMMS) is a self-report tool that evaluates the strength of motivation medical students have for training in medical school. The SMMS consists of three subscales that evaluate different domains of motivation: willingness to sacrifice (i.e. the willingness of a student to sacrifice for his/her medical study), readiness to start (i.e. the readiness and will to enter/continue medical study), and persistence (i.e. the persistence in medical study in spite of unfriendly circumstances during or after the study [21]. Responses are provided on a 5-point Likert scale and subscale scores can range from 5 to 25. Higher values suggest greater motivation to continue training in medical school.

### Data analysis

All data was exported from Google Survey to Excel v365 (Microsoft Corporation, Redmond, WA). All analyses were performed using SPSS v25 (IBM Corporation, Armonk, NY). Descriptive summary statistics were calculated for all student demographics. Normality of data was evaluated using the Shapiro-Wilk test. A *P*-value less than 0.05 was considered significant.

### MBI-SS score calculation

Reverse scoring for the MBI-SS was performed as previously described [22]. Both two- and three-dimensional burnout scores were calculated based on the subjective nature of burnout amongst healthcare professionals [23–25]. The conceptualization of burnout has been subjected to scrutiny over the years. Although the MBI remains the gold standard for measuring subjective burnout, respondents may report burnout differently using one of the three factors. Regardless of these differences, our report shows that burnout can be accurately predicted in our cohort using either the two factor or three factor model. Two calculation methods, the summation (SUM) and average (AVE) method, are two validated techniques to compare burnout scores between respondents. MBI-SS scores were calculated using each method to determine if self-perceived burnout differed using the Student’s t-test for independent groups. The two-dimensional MBI-SS model is the addition of EE and CY, while the three-dimensional model is the addition of all subscales, EE, CY, and AE. No significant difference was noted using either method when comparing subscale scores, in addition to the two- and three-dimensional burnout score. MBI-SS scores reported herein were calculated as means ± standard deviation (SD) using the SUM method.

### Internal consistency of MBI-SS using Cronbach’s alpha

Cronbach’s alpha was used to calculate internal consistency of all MBI-SS items and subscales. Reliability scores of ≥0.5 were considered acceptable with higher scores indicating greater internal consistency [26].

### Factor analysis of MBI-SS using correlation matrix

Statistical data reduction and analysis was performed on the data to explain the correlation between the summated scores in the three subscales (EE, CY, and AE) as previously described [20]. Items with a loading of greater than 0.40 were considered significant. Oblique rotation of the matrix in the factor analysis was performed to account for potential cross-loadings between the MBI-SS subscales. Oblique rotation of the matrix assumed that the 3 MBI-SS subscales were not independent. The analysis in the supplementary file (Additional file 1) shows there is no difference between both the two- and three-dimensional burnout score models.

### Multiple regression of MBI-SS

The association between demographic factors, educational debt, effectiveness of wellness initiatives, the SMMS toolkit, and MBI-SS scores were examined using a series of linear regression models. MBI-SS subscales, two-factor, and three-factor scores were coded as dependent variables. All variables entered the models and beta coefficients with t-values were outputted to assess correlations.

### SMMS score calculation

Reverse scoring for the validated SMMS was performed as previously described [27]. SMMS subscales (sacrifice, readiness to start, and persistence) were summated to develop a three-dimensional model of motivation. Mean ± SD of the summated SMMS score were reported.

## Results

The survey was completed by 273 out of 1264 (21.6%) UICOM students. Response rates by student demographics are shown in Table 1.

**Table 1:** Student demographics, *n* = 273

Variable	n	% of respondents
*Campus*		
Chicago	125	15.8
Rockford	86	38.6
Peoria	62	25.3
*Year of Enrollment*		
M1	88	26.5
M2	54	16.3
M3	79	25.2
M4	52	18.2
*Time Off Before Matriculation (in years)*		
0	112	41.0
1	77	28.2
2	42	15.4
3	17	6.2
4	11	4.0
5	7	2.6
≥ 6	7	2.6
*Gender*		
Male	112	40.9
Female	158	57.7
No Response	3	1.1
*Age Group (in years)*		
21–23	57	20.9
24–26	144	52.7
27–29	51	18.7
30–32	13	4.8
33–35	3	1.1
≥ 36	3	1.1
*Educational Debt (USD)*		
$0	43	15.7
$1 - $100,000	89	32.5
$100,000 - $200,000	68	24.8
$200,000 - $300,000	47	17.2
$300,000 - $400,000	16	5.8
$400,000 - $500,000	6	2.2
≥ $500,000	1	0.4
TOTAL	273	21.5

UICOM medical student characteristics with response rate

A total of 107 (39.2%) students reported self-perceived burnout. Internal reliability (Cronbach’s alpha) coefficients of the MBI-SS subscales were 0.834 for EE, 0.769 for AE, and 0.858 for CY. Minimum acceptable value for reliability coefficient was 0.5 with values greater than 0.75 being preferable [26]. Statistical significance (*P*-value < 0.003) was identified between self-perceived burnout and non-burnout students in all MBI-SS items, in addition to the three subscales. Mean ± SD for all MBI-SS items and subscale scores with reliability coefficients are listed in Table 2.

**Table 2:** Descriptive statistics of MBI-SS with internal consistency, *n* = 273

	Burnout	Non-Burnout	***P***-Value^a^	Cronbach’s Alpha^b^
Emotional Exhaustion	23.23 ± 4.74	14.96 ± 5.71	**0.001**	0.834
ITEM 1	4.59 ± 1.09	2.69 ± 1.40	**0.001**	0.687
ITEM 2	5.0 ± 1.12	3.62 ± 1.56	**0.001**	0.690
ITEM 3	4.91 ± 1.27	3.60 ± 1.59	**0.001**	0.687
ITEM 4	4.11 ± 1.36	2.57 ± 1.33	**0.001**	0.686
ITEM 5	4.64 ± 1.13	2.49 ± 1.42	**0.001**	0.682
Academic Efficacy	24.81 ± 5.35	28.74 ± 3.21	**0.001**	0.769
ITEM 1	4.58 ± 1.29	5.18 ± 0.97	**0.001**	0.723
ITEM 2	3.66 ± 1.52	4.25 ± 1.37	**0.002**	0.725
ITEM 3	4.10 ± 1.55	4.82 ± 1.16	**0.001**	0.722
ITEM 4	4.13 ± 1.51	4.91 ± 1.18	**0.001**	0.730
ITEM 5	4.77 ± 1.14	5.17 ± 0.88	**0.003**	0.724
ITEM 6	3.65 ± 1.25	4.47 ± 1.21	**0.001**	0.725
Cynicism	14.44 ± 5.59	7.59 ± 5.16	**0.001**	0.858
ITEM 1	3.86 ± 1.77	1.75 ± 1.47	**0.001**	0.681
ITEM 2	4.22 ± 1.61	2.34 ± 1.53	**0.001**	0.679
ITEM 3	3.81 ± 1.67	2.03 ± 1.70	**0.001**	0.680
ITEM 4	2.54 ± 1.81	1.51 ± 1.48	**0.001**	0.725

Descriptive statistics of MBI-SS with internal consistency, *n* = 273. ^a^ Independent Student’s t-test for significance and a *P-*value < 0.05 was considered significant. ^b^ Cronbach’s alpha evaluated internal consistency and a value > 0.50 was considered acceptable

To assess the MBI-SS scale items regarding the current study population, factor analyses were run and item loadings were comparable to prior publications using the 15-item MBI-SS [19, 24, 25]. All items were significant. Also, correlations between the MBI-SS subscales were similar to prior publications (See Additional file 1 for factor analysis details).

### Student demographics as predictors of burnout

MBI-SS subscale scores were calculated for student demographics, effect of burnout on desired specialty, and awareness of wellness initiatives (Table 3). High EE, CY, and AE subscale scores were assessed to determine the percent of student demographics affected by high levels of burnout, using one SD above the mean for EE and CY and one SD below the mean for AE as indicators of severe burnout. Significance was identified for self-perceived burnout between years of enrollment (*P*-value = 0.005) gender (*P*-value = 0.001), and effect of desired specialty (*P*-value = 0.001). No significance was noted for self-perceived burnout between campus (*P*-value = 0.177), ethnicity (*P*-value = 0.062), having children (*P*-value = 0.050), being religious/spiritual (*P*-value = 0.162), and awareness of wellness initiatives (*P*-value = 0.362).

**Table 3:** MBI-SS subscale scores and association between self-reported burnout, n = 273

	Emotional Exhaustion		Cynicism		Academic Efficacy			
	Mean ± SD	High EE score n (%)^a^	Mean ± SD	High CY score n (%)^a^	Mean ± SD	High AE score n (%)^a^	Burnout n (%)	χ2-statistic^b^ (***P***-value^c^)
All responses (n = 273)	18.3 ± 6.67	23 (8.4)	10.41 ± 6.29	23 (8.4)	27.11 ± 5.34	27 (9.9)	107 (39.2)	
*Campus*								
Chicago (*n* = 125)	18.56 ± 7.12	13 (10.4)	10.86 ± 6.11	16 (12.8)	27.4 ± 5.4	13 (10.4)	56 (44.8)	3.46 (0.177)
Peoria (*n* = 59)	17.55 ± 6.45	3 (5.1)	9.26 ± 5.84	1 (1.7)	26.66 ± 6.86	8 (13.6)	33 (38.8)	
Rockford (*n* = 85)	18.48 ± 6.19	7 (8.2)	10.67 ± 5.78	6 (7.1)	27.03 ± 5.45	6 (7.1)	18 (30.5)	
*Year of enrollment*								
M1 (*n* = 88)	17.39 ± 6.44	4 (4.6)	8.67 ± 6.07	7 (8)	27.77 ± 4.68	6 (6.8)	26 (29.5)	12.66 (**0.005**)
M2 (*n* = 51)	19.3 ± 6.86	4 (7.8)	10.91 ± 5.9	2 (3.9)	25.65 ± 6.1	10 (19.6)	23 (45.1)	
M3 (*n* = 78)	20.1 ± 6.04	9 (11.5)	12.46 ± 6.40	10 (12.8)	26.68 ± 5.24	7 (9)	42 (53.8)	
M4 (*n* = 52)	16.13 ± 7.08	6 (11.6)	9.85 ± 5.89	4 (7.7)	28.17 ± 5.47	4 (7.7)	16 (30.8)	
*Gender*								
Male (*n* = 110)	17.22 ± 6.63	6 (5.5)	9.81 ± 6.16	7 (6.4)	27.63 ± 5.24	8 (7.3)	30 (27.3)	11.69 (**0.001**)
Female (*n* = 156)	19.07 ± 6.68	17 (10.9)	10.8 ± 6.3	15 (9.6)	26.7 ± 5.46	19 (12.2)	75 (48.1)	
*Ethnicity*								
Asian (n = 59)	18.86 ± 7.14	6 (15.4)	11.02 ± 6.67	6 (15.4)	25.76 ± 5.73	10 (25.6)	27 (45.8)	8.97 (0.062)
Black/AA (n = 12)	16.75 ± 8.79	2 (5.1)	10.17 ± 8.5	3 (7.7)	27.92 ± 4.68	1 (2.6)	5 (41.7)	
Hispanic/Mexican (*n* = 22)	18 ± 6	1 (2.6)	11.45 ± 4.62	1 (2.6)	27.27 ± 5.4	1 (2.6)	7 (31.8)	
White (*n* = 166)	18.13 ± 6.55	13 (33.3)	10.06 ± 6.2	13 (33.3)	27.7 ± 5.05	12 (30.8)	58 (34.9)	
Other (*n* = 14)	19.79 ± 5.62	1 (2.6)	11 ± 5.62	0 (0)	24.93 ± 6.63	3 (7.7)	10 (71.4)	
*Children*								
Yes (*n* = 10)	14.54 ± 5.8	0 (0)	7.91 ± 5.56	1 (10)	27.91 ± 3.88	0 (0)	1 (10)	3.84 (0.050)
No (*n* = 259)	18.46 ± 6.68	23 (8.9)	10.54 ± 6.27	22 (8.5)	27.08 ± 5.4	27 (10.4)	106 (40.9)	
*Religion/Spiritual*								
Yes (*n* = 122)	17.96 ± 6.88	13 (10.7)	10.29 ± 6.2	11 (9)	27.3 ± 5.74	14 (11.5)	44 (36.1)	1.96 (0.162)
No (*n* = 69)	18.67 ± 5.76	5 (7.2)	10.74 ± 5.9	6 (8.7)	26.93 ± 4.82	5 (7.25)	32 (46.4)	
*Effect of desired specialty*								
Yes (*n* = 84)	22.38 ± 5.75	19 (22.6)	13.94 ± 5.55	0 (0)	25.07 ± 5.75	14 (16.7)	60 (71.4)	50.63 (**0.001**)
No (*n* = 184)	16.4 ± 6.24	4 (2.2)	8.76 ± 5.9	7 (3.8)	28.08 ± 4.91	13 (7.1)	47 (25.5)	
*Awareness of wellness initiatives*								
Yes (*n* = 229)	18.23 ± 0.32	20 (8.7)	10.11 ± 0.31	0 (0)	27.21 ± 0.35	24 (10.5)	88 (38.4)	0.83 (0.362)
No (*n* = 39)	18.44 ± 6.74	3 (7.7)	12 ± 7.4	9 (23.1)	26.79 ± 5.02	3 (7.7)	18 (46.2)	

MBI-SS factor analysis loadings, *n* = 273. Items with loading of ≥0.4 were considered significant

MBI-SS subscale correlations, n = 273

^a^ Burnout population mean ± 1 SD. ^b^ Chi-square test for independence. ^c^ *P-*value < 0.05 considered significant

Multiple regression analysis of the two- and three-dimensional MBI-SS models are shown in Table 4. The independent variables (Model 1), out-of-phase (*P*-value = 0.002) in the curriculum, gender (*P*-value = 0.035), the effectiveness of wellness initiatives (*P*-value = 0.006), and the SMMS toolkit (*P*-value = 0.001), were significantly associated with the two-dimensional MBI-SS model. Being out-of-phase (B = − 13.3) in the curriculum, rating the wellness initiatives as effective (B = − 2.8), and high ratings of the SMMS toolkit (B = − 0.5) were negatively associated with two-dimensional MBI-SS model. Gender (B = 3.7), specifically being female, was positively associated with two-dimensional MBI-SS model. The independent variables (Model 2), out-of-phase (*P*-value = 0.001) in the curriculum, the effectiveness of wellness initiatives (*P*-value = 0.048), and the SMMS toolkit (*P*-value = 0.001), were significantly associated with the three-dimensional MBI-SS model. All variables, being out-of-phase (B = − 11.7) in the curriculum, rating the wellness initiatives as effective (B = − 1.9), and high ratings of the SMMS toolkit (B = − 0.3) were negatively associated with three-dimensional MBI-SS model.

**Table 4:** Regression analysis of MBI-SS two- and three-dimensional models for continuous variables, *n* = 273

	Two-Dimensional			Three-Dimensional		
	Unstandardized beta coefficient	t	P-value	Unstandardized beta coefficient	t	P-value
Year of enrollment	−1.107	− 1.007	0.316	− 0.234	− 0.246	0.806
Campus	0.507	0.446	0.656	−0.569	− 0.58	0.563
Out-of-Phase	−13.308	−3.184	**0.002**	−11.676	−3.235	**0.001**
Time off before matriculation	2.896	1.371	0.172	1.103	0.605	0.546
Gender	3.713	2.121	**0.035**	2.601	1.721	0.087
Age	−0.053	−0.122	0.903	0.234	0.628	0.531
Relationship status	−0.249	−0.111	0.912	−1.981	−1.025	0.307
Children currently	1.474	0.306	0.76	0.671	0.162	0.872
Religious/Spiritual	0.794	0.424	0.672	0.783	0.484	0.629
Educational debt	1.463	1.247	0.214	0.762	0.752	0.453
Effectiveness of wellness initiatives	−2.82	−2.808	**0.006**	−1.726	−1.991	**0.048**
SMMS Toolkit	−0.517	−6.322	**0.001**	−0.316	−4.245	**0.001**

The two- and three-dimensional models had a R-Squared of 0.226 and 0.153, respectively. Both models were significant (*P*-value < 0.001)

Multiple regression analysis of MBI-SS subscale scores was evaluated to determine if any significance in the two- and three-dimensional MBI-SS models were due to individual or a combination of subscale scores (Table 5). The independent variables (Model 3), out-of-phase (*P*-value = 0.006) in the curriculum, gender (*P*-value = 0.017), the effectiveness of wellness initiatives (*P*-value = 0.016), and the SMMS toolkit (*P*-value = 0.001), were significantly associated with the EE subscale. Negative associations between being out-of-phase (B = − 6.5) in the curriculum, rating the wellness initiatives as effective (B = − 1.3), and high ratings of the SMMS toolkit (B = − 0.2) were noted with the EE subscale. Again, gender (B = 2.3), especially being female, was positively associated with the EE subscale. The independent variables (Model 4), the effectiveness of wellness initiatives (*P*-value = 0.024) and the SMMS toolkit (*P*-value = 0.001), were significantly associated with the AE subscale. Both rating the wellness initiatives as effective (B = 1.1) and high ratings of the SMMS toolkit (B = 0.2) were positively associated with the AE subscale. The independent variables (Model 5), out-of-phase (*P*-value = 0.002) in the curriculum, the effectiveness of wellness initiatives (*P*-value = 0.005), and the SMMS toolkit (*P*-value = 0.001), were significantly associated with the CY subscale. Being out-of-phase (B = − 6.8) in the curriculum, rating the wellness initiatives as effective (B = − 2.8), and high ratings of the SMMS toolkit (B = − 0.3) were negatively associated with the CY subscale.

**Table 5:** Regression analysis of MBI-SS subscales for continuous variables, *n* = 273

	Emotional Exhaustion			Academic Efficacy			Cynicism		
	Unstandardized beta coefficient	t	P-value	Unstandardized beta coefficient	t	P-value	Unstandardized beta coefficient	t	P-value
Year of enrollment	−0.751	−1.231	0.22	0.874	1.658	0.099	−0.352	−0.629	0.53
Campus	0.374	0.593	0.554	−1.076	−1.977	0.05	0.135	0.233	0.816
Out-of-Phase	−6.497	−2.801	**0.006**	1.632	0.815	0.416	−6.785	−3.192	**0.002**
Time off before matriculation	1.647	1.406	0.162	−1.793	−1.772	0.078	1.251	1.165	0.246
Gender	2.344	2.413	**0.017**	−1.112	−1.327	0.186	1.352	1.519	0.131
Age	0.045	0.189	0.85	0.287	1.387	0.167	−0.094	−0.428	0.669
Relationship status	0.033	0.027	0.979	−1.732	−1.616	0.108	−0.252	−0.222	0.825
Children currently	1.558	0.583	0.56	−0.802	−0.348	0.728	−0.036	− 0.015	0.988
Religious/Spiritual	0.598	0.575	0.566	−0.011	−0.012	0.99	0.203	0.213	0.831
Educational debt	0.623	0.957	0.34	−0.701	−1.248	0.214	0.842	1.411	0.16
Effectiveness of wellness initiatives	−1.351	−2.425	**0.016**	1.094	2.275	**0.024**	−1.454	−2.847	**0.005**
SMMS Toolkit	−0.233	−5.081	**0.001**	0.201	5.525	**0.001**	−0.282	−6.806	**0.001**

The EE, CY, and AE models had an R-Squared of 0.191, 0.227, 0.210. All models were significant (*P*-value < 0.001)

## Discussion

In the current study, burnout was described as ‘feeling exhausted because of study demands, having a cynical and detached attitude toward one’s study, and feeling incompetent as a medical student. High values for EE and CY while low values for AE were considered consistent with burnout. The main findings of this study were the following: the MBI-SS is a validated tool that may be used in place of the MBI- Health Care Survey (HSS) to assess medical student burnout; student demographics like gender, year in the curriculum, and a student's view on wellness initiatives can be used to identify individuals at risk for developing burnout. A new demographic factor that has not been described in the current literature as associated with medical student burnout, out-of-phase medical students, should be considered in assessing medical students at risk for developing burnout.

### MBI-SS prediction of burnout in medical students

Significance between burnout versus non-burnout students was noted for all MBI-SS items, subscales, two- and three-dimensional models. Internal consistency measured using Cronbach’s Alpha was equal or higher than previous burnout studies using the MBI-SS [16, 28, 29]. Several MBI surveys are available to measure burnout in different populations. The Human Services Survey is the most widely used MBI version and primarily examines burnout in health professions, i.e. physicians, nurses, social workers, etc. [22]. Most investigations examining burnout in medical students have used the MBI-HSS given a student’s responsibility of providing care to patients [30]. The MBI-SS, which is an adaptation of the General Survey, was used in this investigation to examine how the rigors of medical education influence burnout, regardless of their interactions with patients.

Our population was comprised of both preclinical and clinical year medical students who had differing levels of experience interacting with patients. Additionally, as student-doctors, the main goal of medical students is to obtain medical knowledge, and apply that knowledge to patients. Conversely, health care professionals are indeed lifelong learners, yet burnout may not stem from their studies, but rather from the rigors of their job. For example, Friedberg et al. noted that physician’s interactions with electronic health records was associated with decreased job satisfaction in some survey respondents [31]. So, yes medical students do have their share of administrative work, but their focus remains gaining knowledge to be competent clinicians, and to do well on exams.

### Factors to predict burnout in medical students

Year of enrollment was found to be associated with burnout. Additionally, the rate of burnout increased until the M3 year and then sharply decreasing during the M4 year. These findings are similar to previous studies looking at the association between year in medical school and perceived burnout [14, 32]. We hypothesize that this association for second-year medical students is related to their preparation for the USMLE Step 1 Board Exam. Historically, the Step 1 score has a high degree of impact upon a medical student’s ability to Match in a competitive specialty or at a competitive program [33] For third year medical students, their focus is centered on NBME Clerkship Examinations, USMLE Step 2 CS and CK, and scheduling sub-internship electives. Again, all these aspects of the medical education impact an individual’s ability to Match in their desired specialty or desired program. Most M4 students, at the time of the study were finished with residency interviews and were awaiting their match results. So, it is likely that much of the stress and anxiety had diminished as M4 students were looking at transitioning into residency and finalizing graduation requirements.

A second demographic factor associated with burnout in our study population was gender, specifically being female in the two-dimensional model and EE MBI-SS subscale. Higher rates of EE in females have been previously shown and with the two-dimensional model only accounting for EE and CY, it is not surprising that the model be used as a predictor of burnout between genders [34].

Yet, this area has proved to be inconsistent, as other studies have found no association between burnout and gender [19, 35, 36]. Studies looking at gender differences in physicians have suggested some of the following: women are more likely to report emotional exhaustion; women may be more likely to report burnout versus their male counter parts; or age may influence expression of burnout, as older physicians are less likely to express their burnout [37–40].

A third demographic factor associated with burnout in our study, are students who are out of phase in their medical education. An out of phase student is any individual that takes a leave of absence during their medical education. This may due to academic reasons, personal reasons, or medical reasons. Students who were out-of-phase in the curriculum were more likely to report burnout in the two- and three-dimensional model, and EE and CY MBI-SS subscales. Lower scores in the AE MBI-SS subscale were not predictive of burnout. This was likely due to a student’s decision to continue their medical education regardless of how many additional years they need to graduate. At our institution, M1 and M2 students have classes scheduled for them, so if for whatever reason they do not complete their required course, they may have to wait until that specific course is offered during the following cycle. For M3 and M4 students, NBME Shelf Examination, USMLE Step Examinations, and residency application deadlines create constraints on the ability for a student to take time off. The authors hypothesize that these constraints may influence burnout in this population of medical students.

### Wellness initiatives

Students that reported that the wellness initiatives were effective had lower levels of burnout in all categories. This finding provides strong evidence that implementing effective wellness initiatives may be sufficient for reducing burnout. Across UICOM, the services students participated the most in were health services (17.1%), meditation (16.6%), and others (8.3%), such as dog therapy, aromatherapy, and art therapy. Other wellness initiatives that students reported as beneficial were access to mental health resources on campus. Studies have shown participation in various interventions can reduce stress and depression [36]. Yet, one aspect that is important is to ensure that students are aware of these services, and that services like mental health services remain anonymous. For example, Dyrbye et al. showed that as many as two thirds of medical students may not seek help due to perceived stigmas associated with doing so [41].

### Strength of motivation for medical students

Students who scored higher on the SMMS reported less burnout. The authors hypothesize that this may be related to a student’s interest in a given specialty. Essentially, they find school work in that area of medicine more rewarding. In our study, students who reported burnout were more likely to consider switching their medical specialties to one they considered ‘less desirable’. This could have serious implications for any specialty that is experiencing decreased residency matriculation, such as general surgery [3]. Of note though, the level of SMMS persistence subscale was similar between both burnout and non-burnout students. This observation may be due to a student’s determination to continue their education regardless of their level of burnout. With the high cost of medical education, and limited options with an incomplete medical degree, students push to simply complete their medical education and enter an area of medicine that is less competitive and of less interest to them.

### Survey response

21.6% of eligible students at the University of Illinois medical school responded to the survey. We acknowledge a degree of selection bias, as our data was gathered via a web-based survey tool. We hypothesize survey fatigue occurred among our study population for at least two reasons. One reason is associated with the number of emails students received daily. Students at the University of Illinois receive emails from the College of Medicine, University of Illinois at Chicago (university wide emails), and site-specific notifications (Chicago, Peoria, Rockford, and Champaign-Urbana). A second aspect associated with the response rate is more specific to each class year. When the survey was sent out, second year medical students were studying for the USMLE Step 1 examination. Third year medical students were focused on scheduling away rotations/sub-internships, USLME Step 2 CK and CS examinations. Lastly, fourth year medical students were focused on Match Day and their subsequent transition into residency.

### Limitations

The study was cross-sectional by design and the relationships described are only associations and merely suggest a potential causal relationship between burnout and student demographics. The survey period may have influenced perceived level of burnout due to the high stress board examinations and upcoming away rotation applications experienced by M2 and M3 students, respectively. A response rate of 21.6% may suggest that the study was selectively completed by students who possessed a strong opinion about burnout and are not reflective of the entire UICOM medical student population [42, 43]. Lastly, co-morbidities such as mental and physical health, substance abuse, and uncontrollable life events were not assessed in the survey, which have been shown to influence burnout in the medical profession [13, 15, 44, 45].

## Conclusion

The 15-item MBI-SS is a validated tool that can assess burnout in medical students. Burnout among UICOM medical students was slightly less than reported national averages. Student demographics: gender, year of enrollment, and being out-of-phase in the curriculum, may be used to identify at-risk groups susceptible to burnout. The implementation of effective wellness initiatives may be sufficient for ameliorating burnout among medical students. We recommend burnout be longitudinally assessed once a year in all medical students to determine the effectiveness of wellness initiatives and make modifications as appropriate. Burnout is a deleterious issue that can negatively impact the healthcare community. Early identification and resolution of the phenomenon can help improve the health outcomes of patients receiving care from medical students and residents progressing through their education.

## Supplementary information

**Additional file 1.**

**Additional file 2.**

## Data Availability

Data is freely available on reasonable request.

## Abbreviations

UICOM: University of Illinois College of Medicine
UI-Rockford: University of Illinois College of Medicine at Rockford
MS#: Medical Student Year 1–4
MBI-SS: Maslach Burnout Inventory-Student Survey
EE: Emotional Exhaustion
CY: Cynicism
AE: Academic Efficacy
SMMS: Strength of Motivation for Medical School
IRB: Internal Review Board
SD: Standard Deviation
B: Unstandardized Beta Coefficient
