# Supplementary material for: Assessment of burnout in medical students using the Maslach Burnout Inventory-Student Survey: a cross-sectional data analysis
Source: BMC Med Educ. 2020 Oct 21;20:376. doi: 10.1186/s12909-020-02274-3 (PMC7579892; doi:10.1186/s12909-020-02274-3)
Supplement: Supplementary file 1 — Additional file 1. [file 12909_2020_2274_MOESM1_ESM.docx]

**Supplement -** **Results and Tables related to Factor Analysis**

MBI-SS factor analysis are summarized in Table A. Item loadings were comparable to prior publications using the 15-item MBI-SS and all items were significant (1-3). Items 1-5 were considered as EE and loaded negatively with AE and positively with CY. Items 6-11 were considered AE and loaded negatively with both EE and CY. Items 12-15 were considered CY and loaded positively with EE and negatively with AE. Correlations between the MBI-SS subscales were similar to prior publications and are shown in Table B. The subscale AE was negatively correlated with the EE and CY subscales while the EE and CY subscales were positively correlated.

Review of factor analysis results shows the loading of the 15-item MBI-SS were similar to previous investigations focusing on medical students (1). EE and CY were found to be positively correlated, while EE and CY were negatively correlated with AE. As expected, EE and CY should both increase in an individual with burnout. In comparison, a student’s AE should decrease with increased values of EE and CY. The negative between burnout and motivation for continued medical education was confirmed using the SMMS toolkit. Significance between burnout and the SMMS was noted between all subscales, in conjunction with the two- and three-dimensional models.

**Table A**. MBI-SS factor analysis loadings, n=273

|  | **Emotional Exhaustion** | **Academic Efficacy** | **Cynicism** |
| --- | --- | --- | --- |
| **Emotional Exhaustion** | | | |
| ITEM 1 | 0.839 | -0.377 | 0.650 |
| ITEM 2 | 0.819 | -0.341 | 0.560 |
| ITEM 3 | 0.828 | -0.309 | 0.606 |
| ITEM 4 | 0.814 | -0.349 | 0.705 |
| ITEM 5 | 0.881 | -0.379 | 0.701 |
| **Academic Efficacy** | | | |
| ITEM 6 | -0.234 | 0.627 | -0.285 |
| ITEM 7 | -0.258 | 0.703 | -0.318 |
| ITEM 8 | -0.236 | 0.698 | -0.239 |
| ITEM 9 | -0.398 | 0.693 | -0.402 |
| ITEM 10 | -0.281 | 0.639 | -0.389 |
| ITEM 11 | -0.340 | 0.790 | -0.358 |
| **Cynicism** | | | |
| ITEM 12 | 0.739 | -0.465 | 0.861 |
| ITEM 13 | 0.764 | -0.474 | 0.903 |
| ITEM 14 | 0.647 | -0.366 | 0.879 |
| ITEM 15 | 0.478 | -0.313 | 0.776 |

**Table B.** MBI-SS subscale correlations, n=273

|  | **Emotional Exhaustion** | **Academic Efficacy** |
| --- | --- | --- |
| **Academic Efficacy** | -0.421 |  |
| **Cynicism** | 0.770 | -0.486 |

**Supplement References**

1. Galán F, Sanmartín A, Polo J, Giner L. Burnout risk in medical students in Spain using the Maslach Burnout Inventory-Student Survey. Int Arch Occup Environ Health. 2011;84(4):453–9.
2. Leibach G, Stern M. Critical Synthesis Package: Strength of Motivation for Medical School Revised (SMMS-R) Questionnaire. MedEdPORTAL Publ. 2014;
3. Portoghese I, Leiter MP, Maslach C, Galletta M, Porru F, D’Aloja E, et al. Measuring burnout among university students: Factorial validity, invariance, and latent profiles of the Italian Version of the Maslach Burnout Inventory Student Survey (MBI-SS). Front Psychol. 2018;9(NOV):1–9.
